# Microdosimetric Modeling of Biological Effectiveness for Boron Neutron Capture Therapy Considering Intra- and Intercellular Heterogeneity in ^10^B Distribution

**DOI:** 10.1038/s41598-017-18871-0

**Published:** 2018-01-17

**Authors:** Tatsuhiko Sato, Shin-ichiro Masunaga, Hiroaki Kumada, Nobuyuki Hamada

**Affiliations:** 1 Japan Atomic Energy Agency (JAEA), Nuclear Science and Engineering Center, Research Group for Radiation Transport Analysis, 2-4 Shirakata, Tokai, Ibaraki, 319-1195 Japan; 2 Particle Radiation Biology, Department of Radiation Life and Medical Science, Research Reactor Institute, Kyoto University, 2-1010 Asashiro-nishi, Kumatori, Sennan, Osaka, 590-0494 Japan; 3 Proton Medical Research Center, University of Tsukuba, 2-1-1 Amakubo, Tsukuba, Ibaraki, 305-8576 Japan; 4 Radiation Safety Research Center, Nuclear Technology Research Laboratory, Central Research Institute of Electric Power Industry (CRIEPI), 2-11-1 Iwado-kita, Komae, Tokyo, 201-8511 Japan

## Abstract

We here propose a new model for estimating the biological effectiveness for boron neutron capture therapy (BNCT) considering intra- and intercellular heterogeneity in ^10^B distribution. The new model was developed from our previously established stochastic microdosimetric kinetic model that determines the surviving fraction of cells irradiated with any radiations. In the model, the probability density of the absorbed doses in microscopic scales is the fundamental physical index for characterizing the radiation fields. A new computational method was established to determine the probability density for application to BNCT using the Particle and Heavy Ion Transport code System PHITS. The parameters used in the model were determined from the measured surviving fraction of tumor cells administrated with two kinds of ^10^B compounds. The model quantitatively highlighted the indispensable need to consider the synergetic effect and the dose dependence of the biological effectiveness in the estimate of the therapeutic effect of BNCT. The model can predict the biological effectiveness of newly developed ^10^B compounds based on their intra- and intercellular distributions, and thus, it can play important roles not only in treatment planning but also in drug discovery research for future BNCT.

## Introduction

Neutron capture therapy using the ^10^B(*n*, *α*) ^7^Li reaction, BNCT, is one of the most effective therapeutic modalities for treating locally invasive malignant tumors thanks to the large neutron-capture cross sections of ^10^B as well as short ranges of their secondary particles. Upon this reaction, 1.77 MeV α particle and 1.02 MeV ^7^Li ion are emitted at the branching ratio of 6.3%, while 1.47 MeV α particle, 0.84 MeV ^7^Li ion, and 0.478 MeV photon are produced in the remaining 93.7% cases. In addition, 0.54 MeV protons emitted from the ^14^N(*n*, *p*)^14^C reaction, recoil protons produced by the ^1^H(*n*,*n*)*p* reaction, photons generated by the ^1^H(*n*,*γ*)^2^H reaction, and contamination photons in the neutron beam also contribute to energy deposition during BNCT. In the treatment planning of BNCT, the absorbed doses deposited by ^10^B(*n*, *α*)^7^Li, ^14^N(*n*, *p*)^14^C, ^1^H(*n*, *n*)*p*, and photons are separately calculated^1,2^, which are referred hereinafter to as boron, nitrogen, hydrogen, and photon components, respectively.

Low energy particles with high linear energy transfer (LET) are dominant contributors to the absorbed doses, except for the photon component. Thus, estimate of their relative biological effectiveness (RBE) is indispensable for the maximal therapeutic efficacy to tumors while sparing normal tissues. Several models have been proposed for evaluating the RBE of high LET charged particles relevant to BNCT^3–6^. However, the therapeutic efficacy depends not only on the RBE but also on the intra- and intercellular heterogeneity of ^10^B distribution. For example, ^10^B-boronphenylalanine (BPA: C_9_H_12_BNO_4_) is more effective for tumor cell inactivation than ^10^B-sodium borocaptate (BSH: Na_2_B_12_H_11_SH) at the same ^10^B concentration owing to cell permeability of BPA^7^. The concept of the compound biological effectiveness (CBE)^8,9^ was then introduced to consider this effect in the BNCT treatment planning. The stochastic nature of the intercellular ^10^B distribution is also expected to influence the therapeutic efficacy^10^ because the surviving fraction (SF) of a cell population generally becomes larger with increasing heterogeneity of absorbed dose in each cell nucleus, particularly at higher doses^11^ Furthermore, the sum of the absorbed dose weighted by fixed RBE or CBE (hereafter, RBE-weighted dose) of each component, which is currently evaluated in the BNCT treatment planning, may not be an adequate index for representing its therapeutic effect, since RBE and CBE vary with the absorbed dose, and the synergistic effect exists in the radiation fields composed by different radiations^12,13^. Thus, the photon dose giving the same therapeutic effect (hereafter, photon-isoeffective dose) was proposed for use in the BNCT treatment planning^14,15^.

Here, we set out to develop a new model for estimating RBE and CBE as well as photon-isoeffective doses of BNCT considering the intra- and intercellular heterogeneity in ^10^B distribution. It is based on the stochastic microdosimetric kinetic (SMK) model^11^, which was developed from the microdosimetric kinetic (MK) model proposed by Hawkins^16^. These models can estimate the cellular SF, not from the profiles of radiation imparting energy such as LET, but from the dose distribution at microscopic sites in the cell nucleus, so-called domains. These models thus consider the synergistic effect intrinsically. In our developed model, dose distributions in domains are calculated by the microdosimetric function^17–19^ implemented in the Particle and Heavy Ion Transport code System PHITS^20^, considering the intracellular ^10^B distributions. In addition, the SMK model was improved to be capable of taking the dose rate effect into account. An advantage of the SMK model over the original MK model is that while the former can fully consider the stochastic nature of the absorbed dose in each cell nucleus, the latter approximates the dose by their mean value. This feature is particularly important when it is applied to BNCT because the greater heterogeneity of the absorbed dose in each cell nucleus are expected for BNCT than for other radiotherapeutic modalities, due to the stochastic nature of the intercellular ^10^B distribution^21^.

Four parameters that express cellular characteristics must be evaluated in the developed model. In this study, their numerical values were determined by the least-square (LSq) fitting of the SF of tumor cells, which we previously determined *in vivo*/*in vitro* experiments of mice exposed to reactor neutron beam with concomitant BPA or BSH treatment at various concentrations^22^. Thus, this paper focuses on the analysis of the therapeutic effect of BNCT, and does not discuss about normal tissue complications. The details of the calculation procedures in the developed model are shown below, and the calculated SF is compared with the corresponding measured data. The influence of the intra- and intercellular heterogeneity in ^10^B distribution on the SF, and the difference in the RBE-weighted and photon-isoeffective doses are discussed.

## Methods

### Principle of the SMK and MK models

The MK model is one of the most successful models to explain the biological effectiveness for the cellular SF. It mathematically interprets the linear-quadratic (LQ) relation of the SF based on the theory of dual radiation action^23^. In the model, the following six basic assumptions were made: (i) a cell nucleus can be divided into multiple domains; (ii) radiation exposure produces two types of DNA damage named lethal and sublethal lesions in cell nuclei; (iii) the number of lethal and sublethal lesions produced in a domain is proportional to the specific energy, *z*, in the domain; (iv) a sublethal lesion is to be repaired, or converted into a lethal lesion via spontaneous transformation or interaction with another sublethal lesion created in the same domain; (v) a domain is to be considered inactivated when an intra-domain lethal lesion is formed; and (vi) a cell is to be considered inactivated when an intranuclear domain is inactivated. Note that the specific energy *z* indicates the energy deposited per mass at a microscopic site. *z* is a stochastic quantity and therefore intrinsically different from the absorbed dose, albeit with the same unit. The definition of specific energy as well as other microdosimetric quantities is summarized in International Commission on Radiation Units and Measurements (ICRU) Report 36^24^.

Based on these assumptions, the SF of a single cell with its nucleus specific energy *z*_n_, ${S}_{{\rm{C}}}({z}_{{\rm{n}}})$, can be calculated by

1 $${S}_{{\rm{C}}}({z}_{{\rm{n}}})=\exp [-({\alpha }_{0}+{\beta }_{0}{\bar{z}}_{{\rm{d}},D}){z}_{{\rm{n}}}-{\beta }_{0}{{z}_{{\rm{n}}}}^{2}],$$

where ${\bar{z}}_{{\rm{d}},D}$ denotes the dose-mean value of specific energy in domain, *z*_d_, per event written as

2 $${\bar{z}}_{{\rm{d}},{\rm{D}}}=\frac{{\int }_{0}^{\infty }{z}_{{\rm{d}}}^{2}{f}_{{\rm{d}},1}({z}_{{\rm{d}}}){\rm{d}}{z}_{{\rm{d}}}}{{\int }_{0}^{\infty }{z}_{{\rm{d}}}{f}_{{\rm{d}},1}({z}_{{\rm{d}}}){\rm{d}}{z}_{{\rm{d}}}},$$

where *f*_d,1_(*z*_d_) is the probability density (PD) of *z*_d_ per event. Note that the term of “event” denotes a single hit of radiation in microdosimetry. The parameters *α*_0_ in Eq. (1) expresses the inactivation sensitivity of domains to lethal lesions, and *β*_0_ represents the interaction probability of two sublethal lesions created in the same domain. In addition to α_0_ and β_0_, the domain radius, *r*_d_, are the free parameters representing the characteristics of the cell type in the MK model. The numerical values of these parameters are independent of the radiation imparting the energy.

Considering the stochastic nature of *z*_n_, the SF of a cell group irradiated with the mean cell-nucleus dose $\overline{{z}_{{\rm{n}}}}$, ${S}_{{\rm{G}}}(\overline{{z}_{{\rm{n}}}})$, can be estimated by

3 $${S}_{{\rm{G}}}(\overline{{z}_{{\rm{n}}}})={\int }_{0}^{\infty }{S}_{{\rm{C}}}({z}_{{\rm{n}}}){f}_{{\rm{n}}}({z}_{{\rm{n}}},\overline{{z}_{{\rm{n}}}}){\rm{d}}{z}_{{\rm{n}}},$$

where ${f}_{{\rm{n}}}({z}_{{\rm{n}}},\overline{{z}_{{\rm{n}}}})$ is the PD of *z*_n_ for the mean cell-nucleus dose $\overline{{z}_{{\rm{n}}}}$, as follows:

4 $${\int }_{0}^{\infty }{z}_{{\rm{n}}}{f}_{{\rm{n}}}({z}_{{\rm{n}}},\overline{{z}_{{\rm{n}}}}){\rm{d}}{z}_{{\rm{n}}}=\overline{{z}_{{\rm{n}}}}.$$

In the original MK model, ${S}_{{\rm{G}}}(\overline{{z}_{{\rm{n}}}})$ is simply approximated by *S*_n_(*z*_n_) by ignoring the stochastic nature of *z*_n_, i.e., substituting the Dirac’s delta-function $\delta (\overline{{z}_{{\rm{n}}}})$ for ${f}_{{\rm{n}}}({z}_{{\rm{n}}},\overline{{z}_{{\rm{n}}}})$ in Eq. (3). In contrast, Eq. (3) is numerically solved in the SMK model because our previous study revealed that the consideration of the stochastic nature of *z*_n_ is of great importance in estimating SF following high-dose, high-LET irradiation^11^. Since the number of events contributing to the energy deposition is expected to follow a Poisson distribution, ${f}_{{\rm{n}}}({z}_{{\rm{n}}},\overline{{z}_{{\rm{n}}}})$ can be calculated by

5 $${f}_{{\rm{n}}}({z}_{{\rm{n}}},\overline{{z}_{{\rm{n}}}})=\sum _{k}\frac{\lambda {(\overline{{z}_{{\rm{n}}}})}^{k}{e}^{-\lambda (\overline{{z}_{{\rm{n}}}})}}{k!}{f}_{{\rm{n}},k}({z}_{{\rm{n}}}),$$

where *f*_n,*k*_(*z*_n_) is the PD of *z*_n_ per *k* events, which can be determined from *f*_n,1_(*z*_n_) and *f*_n,*k*−1_(*z*_n_) using the convolution method as written by

6 $${{f}}_{{\rm{n}},k}({z}_{{\rm{n}}})={\int }_{0}^{{z}_{{\rm{n}}}}{{f}}_{{\rm{n}},1}(z){{f}}_{{\rm{n}},k-1}({z}_{{\rm{n}}}-z){\rm{d}}z.$$

The expected value of the Poisson distribution, $\lambda (\overline{{z}_{{\rm{n}}}})$, can be estimated by

7 $$\lambda (\overline{{z}_{{\rm{n}}}})=\frac{\overline{{z}_{{\rm{n}}}}}{\overline{{{z}}_{{\rm{n}},{\rm{F}}}}},$$

where $\,\overline{{{z}}_{{\rm{n}},{\rm{F}}}}$ is the frequency-mean *z*_n_ per event, which can be calculated by

8 $$\,\overline{{{z}}_{{\rm{n}},{\rm{F}}}}={\int }_{0}^{\infty }{{z}}_{{\rm{n}}}{{f}}_{{\rm{n}},1}({z}_{{\rm{n}}}){\rm{d}}{z}_{{\rm{n}}}.$$

Eqs (1–8) can be numerically solved by determining *f*_d,1_(*z*_d_) and *f*_n,1_(*z*_n_), and thus, their evaluations are pivotal in estimating SF based on the SMK model. The calculation procedures of *f*_d,1_(*z*_d_) and *f*_n,1_(*z*_n_) were established based on the microdosimetric and LET-estimator functions, respectively, implemented in PHITS, assuming that all radiations can penetrate the targets. However, this assumption is not valid for estimating *f*_n,1_(*z*_n_) in the radiation fields of BNCT due to the short ranges of secondary particles produced by neutron capture reactions. In addition, the intracellular heterogeneity of ^10^B distribution influences both *f*_n,1_(*z*_n_) and *f*_d,1_(*z*_d_). Accordingly, a new method was developed here for calculating *f*_d,1_(*z*_d_) and *f*_n,1_(*z*_n_) for BNCT as described in the next subsection.

In our previous study, the overkill effect following high LET irradiation was considered in the SMK model by introducing a free parameter *z*_0_ called “saturation parameter”. In a domain with *z* over *z*_0_, the numbers of lethal and sublethal lesions produced in the domain are assumed to be saturated, and not proportional to *z*. However, the model developed here does not need this parameter because the overkill effect was not observed in the measured SF used for the parameter determination in this study, i.e. the higher *z*_0_ values always provided the better fitting results. This is why the double stochastic microdosimetric kinetic (DSMK) model^11^, which can handle the saturation correction more precisely but with longer computational time, was not employed in this study. Taken together, the SMK and DSMK models have a function to deal with the nontargeted effects^25^, but this function is not used in this study because the nontargeted effects play only a negligible role in cell inactivation at high-dose irradiation where estimation of the therapeutic effect is important.

On the other hand, we improved the SMK model to be capable of considering the dose rate effect, which is very important in the estimate of the therapeutic effect of BNCT due to its relatively longer irradiation time in comparison to other radiotherapeutic modalities. Considering the decrease of sublethal lesions during irradiation, Eq. (1) should be replaced by

9 $${S}_{{\rm{C}}}({z}_{{\rm{n}}})=\exp [-({\alpha }_{0}+{{\beta }}_{0}{\overline{{z}}}_{{\rm{d}},{\rm{D}}}){z}_{{\rm{n}}}-G{\beta }_{0}{{z}_{{\rm{n}}}}^{2}],$$

where *G* is the correction factor of the quadratic coefficient of the LQ relationship^26,27^. The numerical value of *G* can be approximated by

10 $$G=\frac{2}{{({\gamma }_{0}T)}^{2}}[\exp (-{\gamma }_{0}T)-1+{\gamma }_{0}T],$$

where *T* is the irradiation time, and *γ*_0_ is the first order rate constant of repair and spontaneous transformation of sublethal lesions. In this study, *γ*_0_ is regarded as a free parameter.

### Calculation of PD of *z*_d_ and *z*_n_ considering intracellular ^10^B distribution

**Figure 1:**
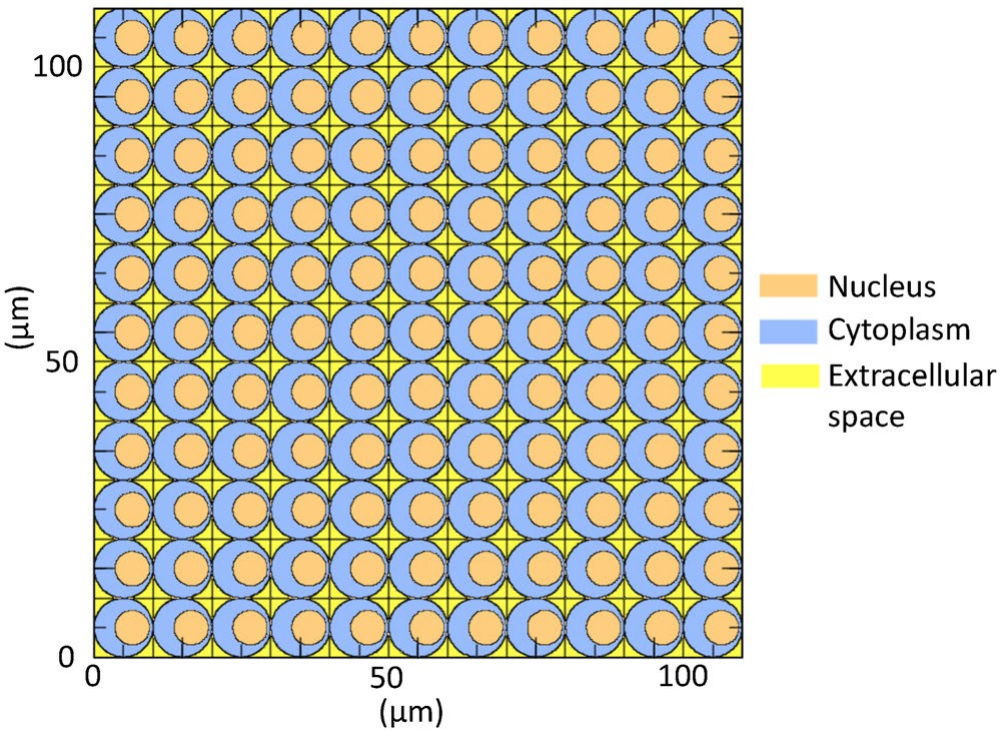
Cross-sectional view of our modeled cellular matrix depicted by PHITS.

In order to estimate *f*_d,1_(*z*_d_) and *f*_n,1_(*z*_n_) in the radiation fields of BNCT, we performed the particle transport simulation in a three-dimensional cellular matrix using PHITS version 2.88. The simulation procedure is similar to that conducted for evaluating the effect of internal exposure^28^. Figure 1 shows a cross-sectional view of our modeled cellular matrix depicted by PHITS. Cells and their nuclei were assumed to be spheres with a radius of 5 μm and 3 μm, respectively, which were the approximated values according to the fluorescence microscopy of SCC VII squamous cell carcinomas used in our previously study^22^. The distance between the centers of a cell and its nucleus is fixed at 1.5 μm, which corresponds to its mean value calculated under the assumption that a cell nucleus is randomly located in the cytoplasm. They were placed in an 11 × 11 × 11 lattice structure, yielding 1,331 cells in the system. The density of cells including their nucleus and extracellular space was set to 1 g/cm^3^, of which composition was assumed to be the same as that used in the dose evaluation of the experimental data, i.e., H (11.1%), C (12.6%), N (2.1%), and O (74.2%) by weight.

In the PHITS simulation for determining *f*_d,1_(*z*_d_) and *f*_n,1_(*z*_n_) for the boron component, secondary charged particles emitted from neutron capture reaction of ^10^B, i.e., an α particle and a ^7^Li ion, were generated to the opposite directions as a Monte Carlo history, using the correlation source generation function of PHITS. The branching ratio of the reaction was considered, though the generation of 0.478 MeV secondary photons was ignored in the simulation because their energy depositions were categorized as the photon component. The PHITS simulations were carried out four times by changing the source localizations, assuming the uniform distribution of ^10^B in the cell nucleus, cytoplasm, extracellular space, or at a boundary between cytoplasm and extracellular space (hereafter, cell surface) of the central lattice. The PD of *z*_n_ per event, *f*_n,1_(*z*_n_), was estimated by calculating the energy deposited in each cell nucleus per Monte Carlo history. In addition, the PD of *z*_d_ per event, *f*_d,1_(*z*_d_), in cell nuclei was calculated using the microdosimetric function in PHITS by changing the domain radii from 0.1 to 0.3 μm by 0.01 μm step. For each condition, Monte Carlo history was set to 1 million, and the PHITS simulation took approximately 15 minutes using a Linux system with 32 CPU cores. Note that the microdosimetric function was developed by fitting the results of track-structure simulation, and thus, it can analytically determine the PD of microscopic sites down to the nanometer scales, considering the dispersion of deposition energies due to the production of δ-rays. Therefore, each domain in cell nucleus was not explicitly specified in the geometry of the PHITS simulation, as shown in Fig. 1.

Considering the intracellular heterogeneity of the ^10^B distribution, *f*_d,1_(*z*_d_) for the entire boron component, ${{f}}_{{\rm{d}},1}^{{\rm{B}}}({z}_{{\rm{d}}})$, can be obtained by averaging the data for each localized source, ${{f}}_{{\rm{d}},1}^{{\rm{B}},{\rm{n}}}({z}_{{\rm{d}}})$, ${{f}}_{{\rm{d}},1}^{{\rm{B}},{\rm{c}}}({z}_{{\rm{d}}})$, ${{f}}_{{\rm{d}},1}^{{\rm{B}},{\rm{s}}}({z}_{{\rm{d}}})$, and ${{f}}_{{\rm{d}},1}^{{\rm{B}},{\rm{e}}}({z}_{{\rm{d}}})$, weighted by their ^10^B fractions as written by

11 $${{f}}_{{\rm{d}},1}^{{\rm{B}}}({z}_{{\rm{d}}})=\frac{{N}_{{\rm{n}}}{{f}}_{{\rm{d}},1}^{{\rm{B}},{\rm{n}}}({z}_{{\rm{d}}})+{N}_{{\rm{c}}}{{f}}_{d,1}^{{\rm{B}},{\rm{c}}}({z}_{{\rm{d}}})+{N}_{{\rm{s}}}{{f}}_{d,1}^{{\rm{B}},{\rm{s}}}({z}_{{\rm{d}}})+{N}_{{\rm{e}}}{{f}}_{{\rm{d}},1}^{{\rm{B}},{\rm{e}}}({z}_{{\rm{d}}})}{{N}_{{\rm{n}}}+{N}_{{\rm{c}}}+{N}_{{\rm{s}}}+{N}_{{\rm{e}}}},$$

where *N* is the relative number of ^10^B distributed in each cell compartment, and their subscripts (i.e., “n”, “c”, “s” and “e”) indicate the data for cell nucleus, cytoplasm, cell surface, and extracellular space, respectively. The total number of ^10^B in the entire region including extracellular space is normalized to 1.0, i.e., *N*_n_ + *N*_c_ + *N*_s_ + *N*_e_ = 1.0. For BPA, we set *N*_n_ = *N*_s_ = 0 because BPA enters cells through amino acid transporters^21,29^, and is assumed here to distribute in the cytoplasm, but not in the cell nucleus. For BSH, we set *N*_n_ = *N*_c_ = 0 because BSH does not pass through the cell membrane^30^. The ratios between the ^10^B concentrations in intra- and extracellular regions were taken from the experimental data for malignant cells, which were approximately 3.2 for BPA and 0.86 for BSH^31^. Then, *N*_c_ and *N*_e_ for BPA were evaluated to be 0.78 and 0.22, respectively, and *N*_s_ and *N*_e_ for BSH were to be 0.48 and 0.52, respectively, considering the volume ratio between intra- and extracellular regions in our model. The PD of *z*_n_ for the entire boron component, ${f}_{{\rm{n}},1}^{B}({z}_{{\rm{n}}})$, can also be determined from an equation similar to Eq. (11) by replacing *f*_d,1_(*z*_d_) by *f*_n,1_(*z*_n_).

To calculate *f*_d,1_(*z*_d_) and *f*_n,1_(*z*_n_) for other dose components, their source terms must be evaluated before conducting the microscopic PHITS simulation because the energy spectra of secondary charged particles depend on the irradiation conditions such as the incident neutron spectrum particularly for the hydrogen component. We therefore performed the macroscopic PHITS simulation to determine the source terms by reproducing the experimental irradiation conditions used in our previous study^21^. In the simulation, a cubic-shaped tumor with volume of 1 cm^3^ was irradiated with neutron beam in the OO-0000-F mode of Kyoto University Research Reactor (KUR)^32^, and spectra of produced charged particles including recoil nuclei were scored for each mother nucleus using the event generator mode^33^. The charged particles emitted only from the ^14^N(*n*,*p*)^14^C reaction were regarded as the nitrogen component, while others were categorized as the hydrogen component. Thus, secondary charged particles emitted from carbon and oxygen were included in the hydrogen component, though their contributions are not so significant. The correlation between recoil proton and carbon ion was considered in simulating the nitrogen component, similarly to the boron component. The spectra of produced electrons were separately scored as the source term for the photon component. Note that the electron spectra produced from the contamination photons in the neutron beam were assumed to be the same as those from photons generated inside the tumor because the spectrum of the contamination photons was not evaluated. Using the evaluated source terms, microscopic PHITS simulations were performed to calculate *f*_d,1_(*z*_d_) and *f*_n,1_(*z*_n_) for the hydrogen, nitrogen, and photon components. The simulation conditions were the same as those used for the boron component calculation except for the source locations, where the charged particles were uniformly generated inside the whole central lattice because the intra- and extracellular heterogeneity of other dose components was not considered in this study.

For the mixed radiation fields of the boron, hydrogen, nitrogen, and photon components, *f*_d,1_(*z*_d_) is simply obtained from the frequency-mean of the data for each component as written by

12 $${{f}}_{{\rm{d}},1}({z}_{{\rm{d}}})=\frac{{\lambda }_{{\rm{B}}}{{f}}_{{\rm{d}},1}^{{\rm{B}}}({z}_{{\rm{d}}})+{\lambda }_{{\rm{H}}}{{f}}_{{\rm{d}},1}^{{\rm{H}}}({z}_{{\rm{d}}})+{\lambda }_{{\rm{N}}}{{f}}_{{\rm{d}},1}^{{\rm{N}}}({z}_{{\rm{d}}})+{\lambda }_{\gamma }{{f}}_{{\rm{d}},1}^{\gamma }({z}_{{\rm{d}}})}{{\lambda }_{{\rm{B}}}+{\lambda }_{{\rm{H}}}+{\lambda }_{{\rm{N}}}+{\lambda }_{\gamma }},$$

where *λ*_B_, *λ*_H_, *λ*_N_, and *λ*_*γ*_ are the number of energy-deposition events, i.e., $\overline{{z}_{{\rm{d}}}}/\overline{{z}_{{\rm{d}},{\rm{F}}}}$, for the boron, hydrogen, nitrogen, and photon components, respectively, and ${{f}}_{{\rm{d}},1}^{{\rm{B}}}({z}_{{\rm{d}}})$, ${{f}}_{{\rm{d}},1}^{{\rm{H}}}({z}_{{\rm{d}}})$, ${{f}}_{{\rm{d}},1}^{{\rm{N}}}({z}_{{\rm{d}}})$, and ${{f}}_{{\rm{d}},1}^{\gamma }({z}_{{\rm{d}}})$ are the PD of *z*_d_ per event for each component. The PD of *z*_n_ for the mixed radiation fields, *f*_n,1_(*z*_n_), can also be determined using an equation similar to Eq. (12) by replacing *f*_d,1_(*z*_d_) by *f*_n,1_(*z*_n_).

It should be mentioned that the cell-nucleus dose for the boron component, ${\overline{{z}_{{\rm{n}}}}}_{{\rm{B}}}$, is different from the value obtained from the kinetic energy released per unit mass, kerma, of the ^10^B(*n*, *α*)^7^Li reaction multiplied with the neutron fluence and the ^10^B concentration in tumor (hereafter kerma dose *D*) due to the heterogeneity of the intracellular ^10^B distribution. To convert the kerma dose of the boron component, *D*_B_, to ${\overline{{z}_{{\rm{n}}}}}_{{\rm{B}}}$, we calculated the ratios between the absorbed doses in cell nucleus for heterogeneous and homogeneous ^10^B distributions based on the PHITS simulation. The evaluated conversion factors, *κ*_B_, were 0.78 for BPA and 0.51 for BSH. Note that *κ*_B_ depends on the distance between the centers of a cell and its nucleus, and they become 0.82 and 0.45 for BPA and BSH, respectively, when we assume concentric spheres of a cell and its nucleus. On the other hand, the mean absorbed doses in cell nucleus for other components were assumed to be the same as their kerma doses in this study because the difference in the material compositions between cell compartments were not considered in our simulation. The conversion factor from the total kerma dose, *D*, to $\overline{{z}_{{\rm{n}}}}$, κ, can be obtained from

13 $$\kappa =({\kappa }_{{\rm{B}}}{D}_{{\rm{B}}}+{D}_{{\rm{H}}}+{D}_{{\rm{N}}}+{D}_{\gamma })/D.$$

The numerical value of *κ* depends on the characteristics of the irradiation field and the ^10^B compound. Using *κ* and *D*, Eq. (3) can be rewritten as:

14 $${S}_{{\rm{G}}}(\kappa ,D)={\int }_{0}^{\infty }{S}_{{\rm{n}}}({z}_{{\rm{n}}}){f}_{{\rm{n}}}({z}_{{\rm{n}}},\kappa D){\rm{d}}{z}_{{\rm{n}}}.$$

### Consideration of intercellular ^10^B distribution

It is expected that the larger the variance of the intercellular ^10^B distribution in tumor cells, the lower the therapeutic efficacy of BNCT. In order to take this effect into account, we assumed that a cell population consists of a number of cell groups having a ^10^B concentration higher (or lower) than the population mean value by a factor of *x*. Under this assumption, *N*_n_, *N*_c_, and *N*_s_ should be multiplied with *x*, and the conversion factor *κ* depends on *x*. Then, the SF for a cell population with kerma dose *D*, *S*_P_(*D*), can be calculated by the summation of the SF of each cell group as written by

15 $${S}_{{\rm{P}}}(D)=\sum _{i}{S}_{{\rm{G}}}(\kappa ({x}_{i}),D){P}_{i},$$

where *P*_*i*_ denotes the probability of cells belonging to group *i*, which satisfies the condition:

16 $$\sum _{i}{x}_{i}{P}_{i}=1,$$

where *x*_*i*_ is the ^10^B concentration factor for group *i*. For continuous intercellular ^10^B distributions, Eq. (15) can be rewritten as:

17 $${S}_{{\rm{P}}}(D)={\int }_{0}^{\infty }{S}_{{\rm{G}}}(\kappa (x),D)p(x)dx,$$

where *p*(*x*) denotes the PD of cells having a ^10^B concentration factor *x*.

In this study, *S*_P_(*D*) for two different types of the intercellular ^10^B distributions were analyzed, which are the double peak distribution with the same probability, i.e., *P*_1_ = *P*_2_ = 0.5, *x*_1_ = 1 − *σ*, and *x*_2_ = 1 + *σ*, and the Gaussian distribution, i.e., *p*(*x*) = $\exp [-{(x-1)}^{2}/2{\sigma }^{2}]/\sqrt{2{\sigma }^{2}\pi }$, by changing their standard deviation *σ* from 0 to 0.6. For *σ = *0, the probability becomes a single peak with *P*_1_ = 1 and *x*_1_ = 1, indicating that the ^10^B concentrations are the same for all cells. The double peak distribution represents the situation where the intake of the ^10^B compounds depends on cell cycle; both BPA and BSH are associated with higher rates of boron uptake at G_2_/M than at G_0_/G_1_ phase^21^.

### Determination of parameters used in the SMK model

The parameters used in the SMK model, i.e., *α*_0_, *β*_0_, *γ*_0_, and *r*_d_ were determined from the LSq fitting of SF that we previously determined in SCC VII murine squamous cell carcinoma^22^. For experiments, tumor cells were inoculated subcutaneously into the left hind legs of syngeneic female C3H/He mice. ^10^B-carrier solutions with BPA concentrations of 250, 500, and 750 ppm, and BSH concentrations of 125, 250, and 375 ppm were prepared, and administrated to the tumor bearing mice in a volume of 0.02 ml/g body weight. The mice were irradiated with neutron beam in the OO-0000-F mode of KUR^32^. The neutron and contaminated photon dose rates in the neutron beam were approximately 0.90 and 0.66 Gy/h, respectively. For both BPA and BSH, the mean ^10^B concentration in the tumor during irradiation was approximately 17, 23, and 26 μg/g with the low, middle, and high ppm, respectively. Note that the actual ^10^B concentrations varied with time after injection very much, and the uncertainties of the mean values are expected to be 20–30%. These experiments were approved by the Committee on Safety and Ethical Handling Regulations for Laboratory Animal Experiments, Kyoto University, and followed institutional guidelines for the care and use of laboratory animals in research. The kerma doses of the experimental data were reevaluated because the kerma factors employed in the former evaluation were different from those used in the PHITS simulation. In addition, the temporal variation in the ^10^B concentration during irradiation was also considered in the reevaluation. The SF of the tumor cells was measured by a clonogenic cell survival assay. The mice without administrating ^10^B compounds were also irradiated with the neutron beam as well as a ^60^Co γ-ray field with dose rate of 2.0 Gy/min to obtain the reference data. More details about the experiments have been described previously^22^.

In the LSq fitting, the chi-square value, *χ*^2^, was calculated by

18 $${\chi }^{2}=\sum _{j=1}^{n}{[\frac{{{S}}_{{j},\exp }({D}_{j})-{S}_{j,{\rm{cal}}}({D}_{j})}{{\rm{\Delta }}{S}_{j,\exp }({D}_{j})}]}^{2},$$

where *S*_*j*,exp_(*D*_*j*_) and Δ*S*_*j*,exp_(*D*_*j*_) are the measured SF and their uncertainties, respectively, for the irradiation condition *j* with the kerma dose *D*_*j*_, *S*_*j*,cal_ is the corresponding SF calculated by our model, and *n* is the number of irradiation conditions, i.e., experimental data points adopted in the fitting. In the calculation of *S*_*j*,cal_, we employed Eq. (15) and set *P*_1_ = 1 and *x*_1_ = 1, i.e., the heterogeneity of the intercellular ^10^B distributions being ignored due to the difficulty in their evaluation during the measurement. Thus, it is ideal to exclude all experimental data with ^10^B administration from the LSq fitting because those data are supposed to be influenced by the heterogeneity. However, the uncertainties of the evaluated fitting parameters obtained only from the experimental data without ^10^B administration were very large, more than 100%, due to the small number of the data points (only 7 points) for four-parameter fitting. We therefore included the experimental data for BSH administration in the LSq fitting because of their lower sensitivity to the heterogeneity in comparison to the BPA data, as discussed later.

The procedure for the LSq fitting was as follows. At first, we determined the numerical values of *α*_0_, *β*_0_, and *γ*_0_ to give the minimum χ^2^ for each *r*_d_ for which *f*_d,1_(*z*_d_) was obtained from the PHITS simulation, based on the Levenberg-Marquardt algorithm. Then, the practical best-fit value of *r*_d_ was determined by searching the smallest χ^2^ among all calculated data. The evaluated parameters are 0.0422 ± 0.0234 Gy^−1^ for *α*_0_, 0.00822 ± 0.00312 Gy^−2^ for *β*_0_, 4.33 ± 3.74 h^−1^ for *γ*_0_, and 0.24 μm for *r*_d_. The accuracy of the fitting was verified by calculating *χ*^2^ per degree of freedom and ${\overline{R}}^{2}$ for the condition, which are 0.816 and 0.960, respectively. Note that ${\overline{R}}^{2}$ is an adjustment for the coefficient of determination, *R*^2^, which becomes close to 1 when a model reproduces the data well. It should be also mentioned that these parameters were not exactly equal to their best-fit values due to the discreteness of *r*_d_. The difference between the evaluated and best-fit values, however, should be trivial due to little dependence of *χ*^2^ on *r*_d_ around 0.24 μm.

## Results and Discussion

### PD of *z*_d_ and *z*_n_

**Figure 2:**
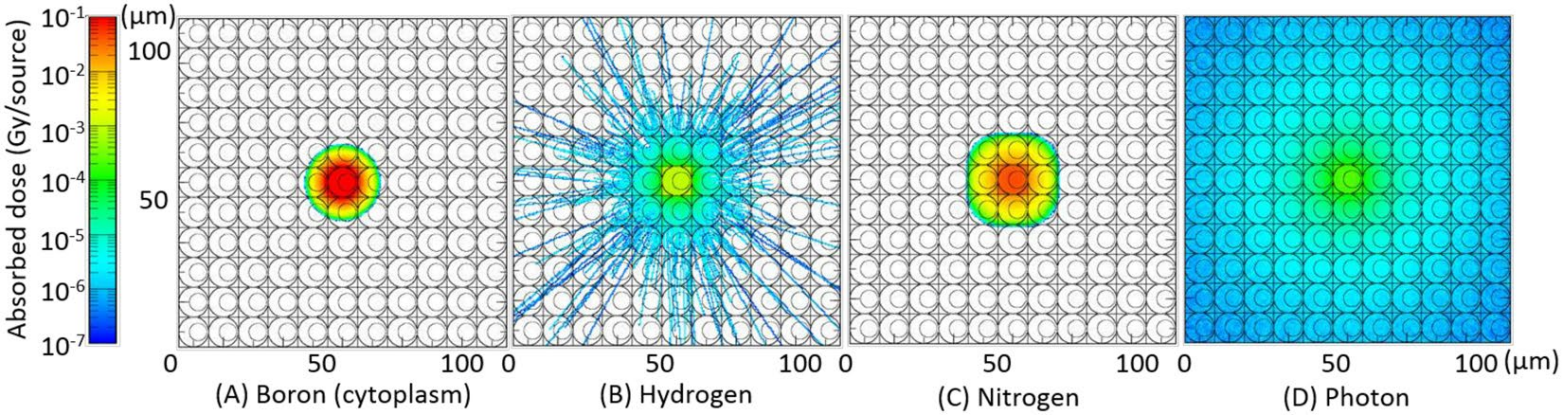
Cross-sectional views of calculated absorbed doses per source generation for each dose component.

Figure 2 shows the cross-sectional views of calculated absorbed doses per Monte Carlo history for each dose component. For the boron component (panel A), the data for the cytoplasmic localization source are depicted. Note that these simulations started with the production of charged particles, so that neutron and photon motions were not simulated. It is evident that while the absorbed doses are concentrated in the central cell or its neighboring cells for the boron and nitrogen components, they are widely spread for the photon component due to longer ranges of secondary electrons. For the hydrogen component, most particles stop very close to the central cell similarly to the nitrogen component, but occasionally travel through several cells because some protons recoiled by high-energy neutrons have an energy in the order of MeV. Note that the range of 1 MeV proton in water is approximately 24 μm.

**Figure 3:**
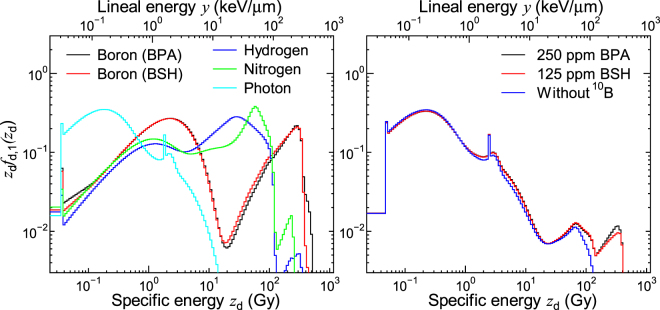
Calculated *z*_d_*f*_d,1_(*z*_d_) for each dose component (left) and some experimental conditions (right). The upper axis denotes the corresponding lineal energy, *y*, which is frequently compared with LET.

Figure 3 shows the *z*_d_*f*_d,1_(*z*_d_) calculated for each dose component and for some experimental conditions. The data for the experimental conditions with higher concentrations of ^10^B compounds are not shown because the calculated PD does not much depend on the ^10^B concentration. The domain radius, *r*_d_, was set to 0.24 μm as its evaluated value in this calculation. The upper axis denotes the corresponding lineal energy, *y*, which is frequently compared with LET. In general, the calculated *z*_d_*f*_d,1_(*z*_d_) is shifted to higher specific energies with an increase in LET of the primary contributing particle for each component. Two broad peaks are observed in the data for the boron component, which are attributed to the contributions from δ-rays and primary ions, respectively. The sharp peak observed in the data for the photon component around 2 Gy is originated from the production of an Auger electron.

**Figure 4:**
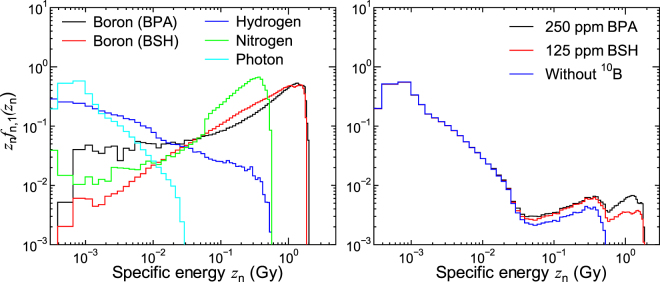
Calculated *z*_n_*f*_n,1_(*z*_n_) for each dose component (left) and some experimental conditions (right).

Figure 4 shows the calculated *z*_n_*f*_n,1_(*z*_n_) for each dose component and for some experimental conditions. Unlike *z*_d_*f*_d,1_(*z*_d_) shown in Fig. 3, the *z*_n_*f*_n,1_(*z*_n_) calculated for BPA and BSH is apparently different from each other; a sudden decrease of *z*_n_*f*_n,1_(*z*_n_) around 0.5 Gy is observed only in the data for BSH. This is because Li ions produced at the cell surface can reach but not penetrate its cell nucleus due to their short range of approximately 4 μm. Note that the events with the highest specific energy, approximately 2 Gy, occur when an α particle or a Li ion penetrates at the center of a cell nucleus. The gap observed in the data for nitrogen component around 0.06 Gy is attributed to the ^14^N(*n*,*p*)^14^C reaction occurring in cell nucleus because such events deposit at least 0.042 MeV, the energy of recoil carbon ion, in cell nucleus.

**Figure 5:**
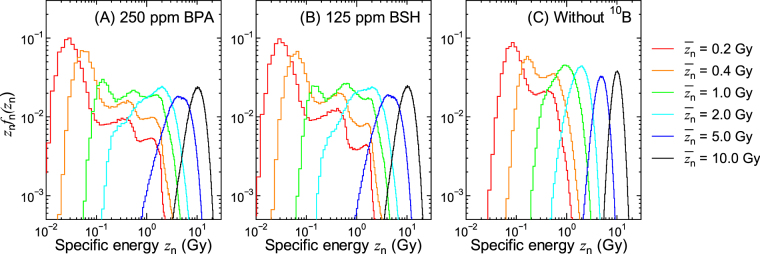
Calculated ${z}_{{\rm{n}}}{f}_{{\rm{n}}}({z}_{{\rm{n}}},\overline{{z}_{{\rm{n}}}})$ for some experimental conditions obtained by numerically solving Eqs (5) and (6)

Figure 5 shows ${z}_{{\rm{n}}}{f}_{{\rm{n}}}({z}_{{\rm{n}}},\overline{{z}_{{\rm{n}}}})$ for some experimental conditions calculated by numerically solving Eqs (5) and (6). The shapes of ${z}_{{\rm{n}}}{f}_{{\rm{n}}}({z}_{{\rm{n}}},\overline{{z}_{{\rm{n}}}})$ are similar to those of the corresponding *z*_n_*f*_n,1_(*z*_n_) for lower $\overline{{z}_{{\rm{n}}}}$, while they become closer to the Gaussian distribution with increasing $\overline{{z}_{{\rm{n}}}}$. It is evident from the graphs that the administration of ^10^B enlarges the variance of the PD even for the highest $\overline{{z}_{{\rm{n}}}}$, indicating higher intercellular dose heterogeneity. Noteworthy is that the intercellular dose heterogeneity becomes even higher when the intercellular ^10^B distribution is considered.

### Surviving fraction

**Figure 6:**
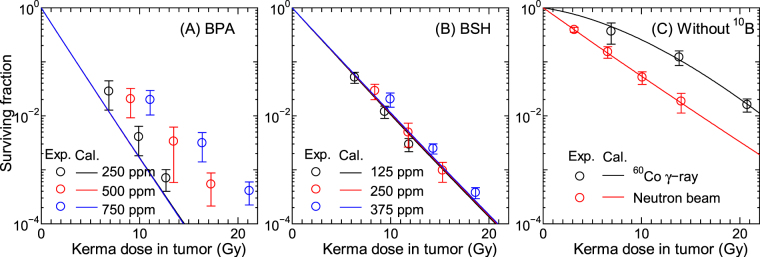
Measured and calculated SF for SCC VII squamous cell carcinomas administrated with (**A**) BPA, (**B**) BSH, and (**C**) without ^10^B compound, plotted as a function of total kerma dose in the tumor including the boron component. Those cells were inoculated into mice and irradiated by neutron beam of KUR except for the ^60^Co γ-ray data shown in Panel (C). The heterogeneity of the intercellular ^10^B distribution is not considered in this calculation.

Figure 6 shows the measured and calculated SF for the tumor cells with administrated BPA (pane1 A), BSH (pane1 B) or without ^10^B compound (panel C), plotted as a function of the total kerma dose in the tumor including the boron component. The calculated data were obtained from Eq. (15) by setting *P*_1_ = 1 and *x*_1_ = 1, i.e., the heterogeneity of the intercellular ^10^B distribution was not considered in this calculation. The agreements between the measured and calculated SF are quite satisfactory for BSH administrated and without ^10^B compound cases, which were used in the LSq fitting. On the other hand, our calculation underestimates the experimental data for BPA cases particularly at higher ^10^B concentrations. In addition, the dependence of the SF on the ^10^B concentration is less significant in our calculated data. These disagreements are probably attributable to the ignorance of the heterogeneity of the intercellular ^10^B distribution as discussed later. At the same kerma dose, the calculated SF for BPA cases are smaller than those for BSH cases because of the larger conversion factor from the kerma dose *D* to the mean cell-nucleus dose $\overline{{z}_{{\rm{n}}}}$, owing to cell permeability of BPA.

**Figure 7:**
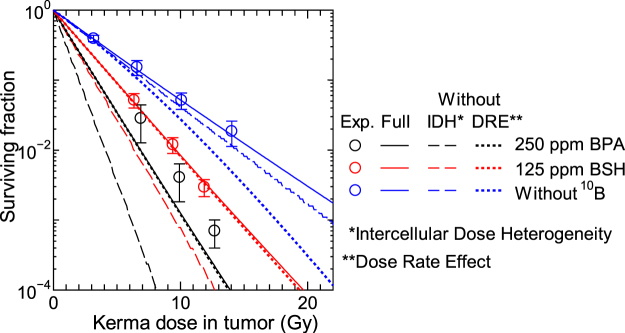
Calculated SF without considering the intercellular dose heterogeneity (IDH) or the dose rate effect (DRE), in comparison with the corresponding data with full consideration. Corresponding experimental data are also plotted in the graph.

Except for the photon data, the *β* terms of the measured SF shown in Fig. 6 are very close to 0 or even negative, and our calculation can reproduce this tendency very well even using the same *β*_0_ parameter for all radiations. This is predominantly because our calculation considered the intercellular dose heterogeneity, which increases the SF for high-dose and high-LET irradiations. In addition, consideration of the dose rate effect also reduces *β* particularly for the data with longer irradiation time as indicated by Eq. (10). For example, the irradiation time *T* is roughly 9 hours and *G* calculated by Eq. (10) is approximately 0.05 for the highest-dose neutron beam irradiation without ^10^B compound. For quantitative discussion, Fig. 7 shows the SF calculated without considering the intercellular dose heterogeneity or the dose rate effect, in comparison with the corresponding data with full consideration. It is evident from the graph that the consideration of the intercellular dose heterogeneity increases the SF very much particularly for ^10^B administrated cases because of their larger heterogeneity. On the other hand, the dose rate effect has little influence on the calculated SF for the ^10^B administrated cases because of two reasons; it becomes less significant for higher dose rates, and it changes only quadratic coefficient of the LQ relationship as written in Eq. (9), while those data are predominantly determined by the linear coefficient. These tendencies indicate that the consideration of the intercellular dose heterogeneity is indispensable to the estimate of the therapeutic effect of BNCT, while that of the dose rate effect is desirable in the estimate of normal tissue complications with lower ^10^B concentration.

**Figure 8:**
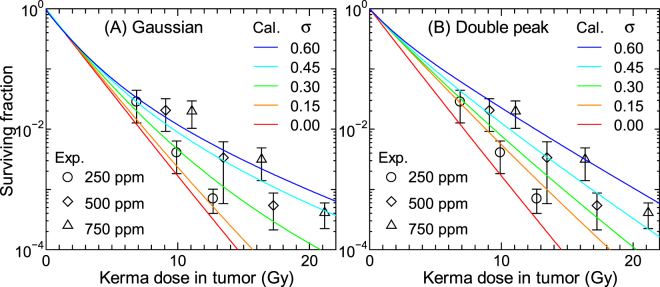
Calculated SF for 250 ppm BPA (17 μg/g) obtained under assumption that the intercellular heterogeneity of ^10^B concentrations can be expressed by the (**A**) Gaussian or (**B**) double-peak distributions with the standard deviation of *σ*. The corresponding experimental data including those for other ^10^B concentrations are also shown in the graph.

**Figure 9:**
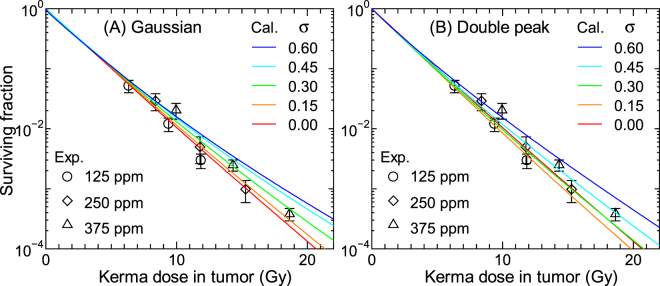
Calculated SF for 125 ppm BSH (17 μg/g) obtained under assumption that the intercellular heterogeneity of ^10^B concentrations can be expressed by the (**A**) Gaussian or (**B**) double-peak distributions with the standard deviation of *σ*. The corresponding experimental data including those for other ^10^B concentrations are also shown in the graph.

Figures 8 and 9 show the SF calculated for 250 ppm BPA and 125 ppm BSH, respectively, assuming that the intercellular heterogeneity of ^10^B concentrations can follow the Gaussian or double-peak distributions with the standard deviation of *σ*. The corresponding experimental data including those for other ^10^B concentrations are also shown in the figures. The data calculated for other ^10^B concentrations are nearly comparable to the plotted data with the same *σ*, similarly to the data shown in Fig. 6. In general, the calculated SF increases with increasing *σ* particularly for the BPA data at higher doses because the intercellular dose heterogeneity becomes greater. The BSH data are less sensitive to *σ* than the BPA data because a large fraction of BSH localizes in the extracellular region whose ^10^B concentration is not affected by *σ*. The SF calculated for the same *σ* assuming the Gaussian and double-peak distributions agrees fairly well with each other. Considering the quite different shapes between the Gaussian and double-peak distributions, this tendency indicates that the intercellular ^10^B distribution can roughly be represented only by their standard deviation, irrespective of the form of the distribution.

As aforementioned, our calculation without considering the intercellular ^10^B distribution underestimates the measured SF of cells administrated with BPA particularly at higher concentrations. In addition, the calculated SF for higher BSH concentrations is also smaller than the corresponding experimental data to some extent. These tendencies can be explained under assumption that *σ* of the intercellular ^10^B distribution increases with an increase in its mean value, since the calculated data for higher *σ* agree with the experimental data at the higher ppm, as shown in Fig. 8. This assumption can be interpreted biologically if the maximum capacity for intake of ^10^B compounds would vary with each cell, such that some cells having less capacity might not incorporate sufficient ^10^B compounds than others when ^10^B compounds are administrated at a higher concentration. However, the relation between the variance and mean of the intercellular ^10^B distribution has not been investigated, warranting further studies to clarify this issue. It should be mentioned that cell cycle plays a very important role in determining the intercellular ^10^B distribution^21^, and thus, the consideration of the cell cycle dependence of the radiation sensitivity is also desirable in our model for more precise estimation of SF.

### RBE and CBE

**Figure 10:**
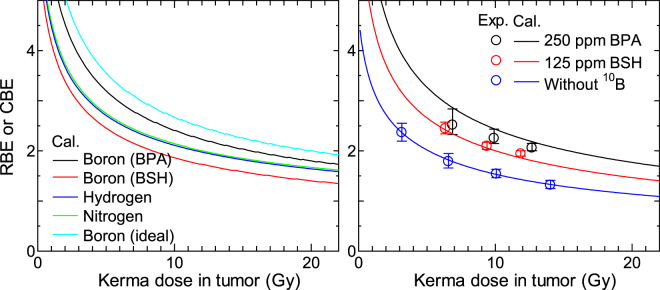
Calculated RBE or CBE for each dose component (left) and for some experimental conditions (right) as a function of the total kerma dose in tumor including the boron component. CBE for an ideal ^10^B compound that can be homogeneously distributed inside cell is also drawn in the left panel.

Figure 10 shows the RBE or CBE calculated for each dose component and for some experimental conditions as a function of the total kerma dose in the tumor including the boron component. The heterogeneity of the intercellular ^10^B distribution was not considered in this calculation. CBE for an ideal ^10^B compound that can be homogeneously distributed inside cell including its nucleus is also drawn in the left panel. As for the terminological distinction between RBE and CBE, the latter is used only for expressing the biological effectiveness of the boron dose component in this paper because the contributions from other dose components should be excluded in the estimate of its numerical value, i.e. the biological effectiveness directly deduced from the experimental conditions is not CBE even for ^10^B administrated cases.

These data were obtained from the equation:

19 $${\rm{RBE}}\,{\rm{or}}\,{\rm{CBE}}=\frac{[-{\alpha }_{\gamma }+\sqrt{{\alpha }_{\gamma }^{2}-4{\beta }_{0}\,\mathrm{ln}(S)}]}{2{\beta }_{0}D},$$

where *S* is the SF for each dose component or experimental condition for the kerma dose *D*, and *α*_γ_ is the *α* term of the SF for the ^60^Co γ-ray irradiation, which was evaluated to be 0.0634 Gy^−1^ from Eq. (1). This equation implies that the SF for the γ-ray irradiation can be simply expressed by the LQ model without considering the stochastic nature of *z*_n_. Experimentally determined RBE shown in the right panel of Fig. 10 were also obtained from Eq. (19), considering the dose rate effect.

It is evident from the graphs that the calculated RBE and CBE decrease with increasing the kerma dose because the *β* term is not clearly observed in the calculated SF except for γ-ray irradiation due to the greater intercellular dose heterogeneity as discussed above. The dose rate effect also reduces the RBE and CBE for the experimental conditions. The CBE for BPA and BSH was smaller than that for the ideal ^10^B compound by factors of approximately 1.2 and 1.8, respectively. This tendency is attributed to *κ*_B_ < 1 for both BPA and BSH owing to their impermeability of cell nucleus. Thus, the therapeutic effect of BNCT would be much higher if a ^10^B compound permeating cell nucleus could be developed. The lower RBE for the experimental condition without ^10^B is due to the longer irradiation time as well as the higher dose fraction of the photon component; approximately 40% of the dose derived from the photon component when ^10^B was absent, whereas the fractions were generally less than 10% when ^10^B was administrated.

Table 1 summarizes the calculated RBE or CBE at certain levels of SF – 50%, 10%, and 1% – for each dose component and experimental condition. As expected from Fig. 10, RBE and CBE decrease with decreasing SF. The RBE values of the experimental conditions for BPA and BSH cases are higher than the corresponding CBE values of the boron components, due to the existence of the synergetic effect in the experimental conditions. It should be mentioned that the kerma dose in the blood, which can be estimated from the ^10^B concentration in the blood instead of tumor, is generally calculated as the physical dose in the BNCT treatment planning. Thus, the calculated CBE data should be multiplied with the ratio of the ^10^B concentrations in tumor and blood when they are used for estimating the therapeutic effect.

**Table 1:** RBE or CBE calculated at certain levels of SF – 50%, 10%, and 1% – for each dose component and experimental condition.

	RBE_50_ or CBE_50_	RBE_10_ or CBE_10_	RBE_1_ or CBE_1_
**Dose component**			
Boron (BPA)	5.49	3.60	2.74
Boron (BSH)	3.55	2.33	1.76
Hydrogen	3.95	2.72	2.16
Nitrogen	4.07	2.78	2.21
Boron (ideal)	6.56	4.30	3.25
**Experimental condition**			
BPA 250 ppm	5.79	3.74	2.81
BPA 500 ppm	5.80	3.75	2.82
BPA 750 ppm	5.79	3.75	2.81
BSH 125 ppm	4.09	2.65	1.99
BSH 250 ppm	4.05	2.62	1.97
BSH 375 ppm	4.02	2.60	1.96
without ^10^B	2.59	1.69	1.25

**Figure 11:**
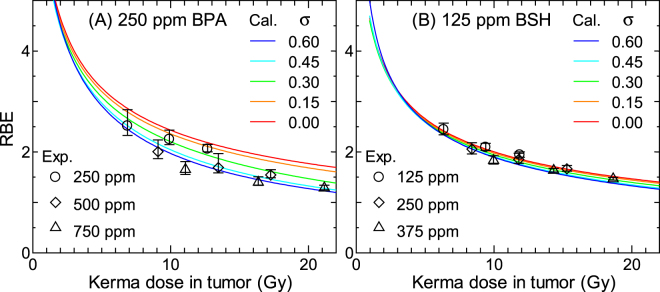
Calculated RBE for (**A**) 250 ppm BPA and (**B**) 125 ppm BSH considering the intercellular heterogeneity in ^10^B distributions. The Gaussian distributions with the standard deviation σ were assumed in the calculation. The corresponding experimental data including those for other ^10^B concentrations are also shown in the graphs.

Figure 11 shows the calculated RBE for 250 ppm BPA and 125 ppm BSH considering the intercellular heterogeneity in ^10^B distributions. The Gaussian distributions with the standard deviation σ were assumed in the calculation. The corresponding experimental data including those for other ^10^B concentrations are also shown in the graphs. As expected from Figs 8 and 9, the calculated RBE decreases more dramatically with increasing the kerma dose at higher σ, particularly when BPA is administered. This tendency suggests that reduction of the variance of the intercellular heterogeneity in ^10^B distributions is important to maintain the higher therapeutic efficacy of high dose irradiation, particularly for BPA.

### RBE-weighted dose and photon-isoeffective dose

**Figure 12:**
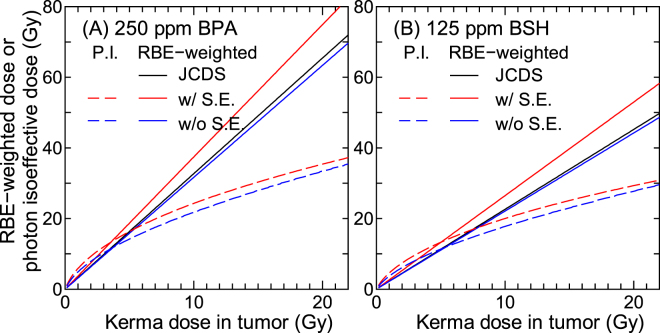
Calculated RBE-weighted doses and photon-isoeffective (P.I.) doses for (**A**) 250 ppm BPA and (**B**) 125 ppm BSH. Red and blue lines denote the data calculated with and without considering the synergetic effect (S.E.), respectively. The RBE-weighted doses estimated from the RBE and CBE values adopted in JCDS are also drawn in the graphs.

Figure 12 shows the calculated RBE-weighted doses and photon-isoeffective doses for 250 ppm BPA and 125 ppm BSH. As aforementioned, the term of “RBE-weighted dose” used for BNCT implies that RBE or CBE is independent of dose and dose rate. In this study, RBE and CBE at 10% SF, i.e., RBE_10_ and CBE_10_ shown in Table 1, were adopted for estimating the RBE-weighted doses, and are thus proportional to the dose. In contrast, the photon-isoeffective doses were estimated from the dose-dependent RBE and CBE as shown in Fig. 10. In both cases, we evaluated the data with or without considering the synergetic effect, i.e., the total dose multiplied with CBE for the experimental condition, or the sum of the doses for each component multiplied with their own RBE or CBE. The RBE-weighted doses that were estimated from the RBE and CBE values adopted in the computational dosimetry system named JCDS^1,34^ are also drawn in the graphs, where CBE for the boron component were 3.8 and 2.5 for BPA and BSH, respectively, and RBE for both hydrogen and nitrogen components were 2.5. Note that JCDS was applied to the BNCT treatment planning of clinical trials conducted in Japan Atomic Energy Agency (JAEA).

The photon-isoeffective dose is higher than RBE-weighted doses at lower kerma dose, whereas the reverse is true at higher kerma dose. The crossing point indicates the kerma dose to give 10% SF. The difference between the RBE-weighted and photon-isoeffective doses becomes larger as the kerma dose increases. Thus, the dose dependence of RBE and CBE should be taken into account in the BNCT treatment planning particularly for high dose irradiation, otherwise the therapeutic effect in comparison to photon therapy would be overestimated. This tendency confirms the conclusion previously obtained from other studies^14,15^, where the photon-isoeffective doses derived from tumor control and normal tissue complication probabilities should be lower than the corresponding RBE-weighted doses, from the viewpoint of microdosimetry. The consideration of the synergetic effect enlarges both RBE-weighted and photon-isoeffective doses because RBE for the experimental conditions is higher than CBE or RBE for each dose component, as shown in Table 1.

## Conclusions

We have here developed a model for estimating the biological effectiveness of BNCT considering the intra- and intercellular heterogeneity in ^10^B distribution on the basis of the SMK model. The synergetic effect of mixed radiation fields can also be taken into account in the model similarly to other cell inactivation models proposed for BNCT^14^. Four free parameters exist in the model, and in this study, their numerical values were determined from the LSq fitting of the SF of tumor cells obtained from our previously reported *in vivo*/*in vitro* measurements using mice administrated with BPA or BSH. Our model can satisfactorily reproduce the measured SF as well as their associated RBE and CBE in various experimental conditions. Our model quantitatively verified that the considerations of the synergetic effect and the dose dependence of RBE and CBE are very important in the estimate of the therapeutic effect of BNCT. The model is planned to be implemented into a research version of our developing treatment planning system, Tsukuba plan^35^, in the near future. For that purpose, the SMK model parameters not only for tumor but also for normal tissue cells must be evaluated because the estimate of normal tissue complications is as important as that of the therapeutic effect in the treatment planning, particularly in the determination of irradiation time. Note that the applications of our model to the estimate of normal tissue complications are limited to those attributable to cell inactivation, such as acute skin reactions^36^.

A unique feature of our model is that it can predict the biological effectiveness of newly developed ^10^B compounds based on their intra- and intercellular heterogeneity. For example, our model suggests that the realization of an ideal ^10^B compound that is homogeneously distributed in the whole cell would enlarge the biological effectiveness by factors of approximately 1.2 and 1.8 in comparison to those of BPA and BSH, respectively. The intercellular homogeneity is also expected to be important to keep the higher therapeutic effect of high dose irradiation, otherwise CBE suddenly drops with increasing dose. Owing to this feature, our model can play important roles not only in the treatment planning but also in developing new ^10^B compounds used for future BNCT. More precise measurement of the intra- and intercellular ^10^B distributions is the key issue for applying our model to the drug discovery research.
